# Family medicine departments and healthcare centres during COVID-19 pandemic in Turkey

**DOI:** 10.1080/13814788.2020.1789095

**Published:** 2020-07-17

**Authors:** Mustafa Kursat Sahin, Gulay Sahin

**Affiliations:** a School of Medicine, Department of Family Medicine, Ondokuz Mayıs University, Samsun, Turkey; b Kadifekale Family Health Center, Samsun, Turkey

I read your article titled ‘Family medicine in times of “COVID-19”: a generalists’ voice’ with great interest [1]. We want to mention that there are generally similar and some differences in family medicine during the COVID-19 pandemic in Turkey. Nearly all family physicians worked during the pandemic in Turkey. Family medicine residents and scientists worked at COVID-19 units, services or intensive care units. During this period, family medicine residents’ training was suspended temporarily. Thesis exams and graduations of family medicine residents were postponed. Some family physicians, residents and scientists were infected. Unfortunately, there have been colleagues who died. There are no exact figures on how many family physicians were infected or died.

The government declared a ban on going out of the house for people who were over 65 years old, had chronic diseases or were under 20 years old. Medication for chronic diseases was taken directly from pharmacies without prescription if patients had a chronic disease report. Appointment hours were determined and applied for child vaccinations. Other patients visited Family Health Centres (FHCs), routinely. The Ministry of Health (MoH) provided all personal protective equipment to all FHCs. Triage was performed in FHCs and then patients were evaluated in rooms that were specially prepared for possible COVID-19 cases. Patients suspected of COVID-19 were transported to hospitals by ambulance or their own vehicles. All COVID-19 treatment was organised in hospitals. Family physicians phoned for follow-up symptoms about COVID-19 if their patients were possible cases, positive cases, discharged from hospital, from foreign countries, or Umrah. The family physician was not informed when anyone hospitalised the patient. The COVID-19 guideline was published and revised by the Coronavirus Scientific Advisory Board, which was established from the MoH [2]. No family medicine scientist was invited to this board. Also, issues related to family medicine were not included in the guidelines published by the MoH. Additional money payments were made to healthcare professionals working in Covid-19 pandemic hospitals. However, no additional payment was made to family physicians who worked in FHCs. Also, the appointment system is not routinely implemented in family medicine in Turkey. Family physicians continued to work between 08.00 and 17.00, routinely.

Family medicine should play an essential role in the normalisation process relating to COVID-19 in Turkey. Many patients staying at home will likely apply to health care centres. Most individuals over the age of 65 years with chronic diseases will need to be monitored. At the same time, new mental health diseases or existing mental health diseases are expected to worsen. During this period, the possibility of transmission through the appointment system and telemedicine can be minimised. It would be appropriate to apply rapid diagnostic tests in FHCs because these centres are more accessible than hospitals. Also, it is very crucial to include family physicians in research studies to find answers for questions related to COVID-19. Departments will play an essential role in this. Conclusively, family physicians’ opinions and suggestions will be needed for all kinds of patient and disease process solutions. However, nothing will be the same, even family medicine.
